# On Death from Cold

**Published:** 1868-12

**Authors:** 


### ARTICLE VII.

### *On Death from Cold.*

"Physiological Studies upon the Effects of Congelation on the Animal Economy." By Luigi De Crecchio, Prof. of Medical Jurisprudence in the Royal University of Naples.

The principal conclusions arrived at by M. Crecchio, in his study of the effect of congelation upon the animal economy, are:

1st. It is impossible to fix a degree of cold, always and absolutely mortal. The degree at which death takes place depends upon the particular and individual circumstances of the special case.

2d. Cold acts by contracting the smaller vessels and by forcing the blood accumulated in them into the larger ones, thus producing anæmia of the frozen part. Occasionally, however, and especially when the action of the cold is not continuous, the vessels dilate through loss of their contractility, and the blood they contain is arrested and the part assumes an obscure red or bluish color.

3d. Blood freezes between a half and one degree below zero (centigrade.) Then it is of vermilion color. Coagulated blood, when frozen immediately, loses the property of coagulation.

4th. The alteration of the blood does not in reality take place during the freezing, but during the thawing process. It does not consist in the rupture of the cell-walls, and the escape of the contents of the globules by this means ; their issue takes place by exosmose, the walls of the globules remaining intact.

5th. Blood removed from the influence of the vessels and life, is altered by cold in a few seconds. If inclosed in the vessels, whether life exist or not, the alteration requires a longer time—a half-hour at least. Moreover, if two animals—the one living, the other dead—are exposed for the same period to congelation, the alteration in the blood will always be greater in the one that was dead before the experiment.

6th. The bloodvessels, it would seem, have the faculty of protecting, up to a certain point, the integrity of the globules against the action of cold ; for while blood, frozen immediately after its withdrawal from the vessels, becomes altered, icicles of that fluid taken from the vessels and allowed to thaw, under the microscope present the globules in the normal state.

7th. Freezing and thawing, whether they take place gradually or very rapidly, produce an alteration of the blood always identical in every respect.

8th. The degree of cold may be sufficient to produce paralysis of the part, and yet not suspend the circulation, at least in vessels of a certain calibre. The arrest of circulation in these latter, is rather the consequence of the action produced upon the enervation than the direct and immediate result of the freezing.

9th. Parts frozen are no longer susceptible of electric stimulus; they are in a state of gangrene. An animal that is frozen dies.

10th. Partial freezing is fatal, when the part frozen is not separated from the rest of the body—not because the blood altered by the cold enters the current of circulation and there causes a sort of infection, but because the materials resulting from the gangrene are absorbed and produce death.

11th. Complete or incomplete freezing kills by congestion, more or less grave, of the internal organs, or by producing stupor or paralysis of the nervous system—more frequently by the two causes combined.

12th. Cadaveric rigidity remains a long time after death from freezing.—*Archives de Physiologie Normale et Pathologiqut. Boston Medical Journal.*

